# HLA-Associated Immune Pressure on Gag Protein in CRF01_AE-Infected Individuals and Its Association with Plasma Viral Load

**DOI:** 10.1371/journal.pone.0011179

**Published:** 2010-06-17

**Authors:** Goragoch Gesprasert, Nuanjun Wichukchinda, Masahiko Mori, Teiichiro Shiino, Wattana Auwanit, Busarawan Sriwanthana, Panita Pathipvanich, Pathom Sawanpanyalert, Toshiyuki Miura, Prasert Auewarakul, Arunee Thitithanyanont, Koya Ariyoshi

**Affiliations:** 1 Department of Microbiology, Faculty of Science, Mahidol University, Bangkok, Thailand; 2 Department of Medical Sciences, National Institute of Health, Ministry of Public Health, Nonthaburi, Thailand; 3 Institute of Tropical Medicine (NEKKEN), Nagasaki University, Nagasaki, Japan; 4 AIDS Research Center, National Institute of Infectious Diseases, Tokyo, Japan; 5 Day Care Center, Lampang Hospital, Lampang, Thailand; 6 Advanced Clinical Research Center, Institute of Medical Science, University of Tokyo, Tokyo, Japan; 7 Department of Microbiology, Faculty of Medicine Siriraj Hospital, Mahidol University, Bangkok, Thailand; 8 Global COE Program, Nagasaki University, Nagasaki, Japan; Karolinska Institutet, Sweden

## Abstract

**Background:**

The human leukocyte antigen (HLA)-restricted cytotoxic T-lymphocyte (CTL) immune response is one of the major factors determining the genetic diversity of human immunodeficiency virus (HIV). There are few population-based analyses of the amino acid variations associated with the host HLA type and their clinical relevance for the Asian population. Here, we identified HLA-associated polymorphisms in the HIV-1 CRF01_AE Gag protein in infected married couples, and examined the consequences of these HLA-selected mutations after transmission to HLA-unmatched recipients.

**Methodology/Principal Findings:**

One hundred sixteen HIV-1-infected couples were recruited at a government hospital in northern Thailand. The 1.7-kb *gag* gene was amplified and directly sequenced. We identified 56 associations between amino acid variations in Gag and HLA alleles. Of those amino acid variations, 35 (62.5%) were located within or adjacent to regions reported to be HIV-specific CTL epitopes restricted by the relevant HLA. Interestingly, a significant number of HLA-associated amino acid variations appear to be unique to the CRF01_AE-infected Thai population. Variations in the capsid protein (p24) had the strongest associations with the viral load and CD4 cell count. The mutation and reversion rates after transmission to a host with a different HLA environment varied considerably. The p24 T242N variant escape from B57/58 CTL had a significant impact on the HIV-1 viral load of CRF01_AE-infected patients.

**Conclusions/Significance:**

HLA-associated amino acid mutations and the CTL selection pressures on the p24 antigen appear to have the most significant impact on HIV replication in a CRF01_AE-infected Asian population. HLA-associated mutations with a low reversion rate accumulated as a footprint in this Thai population. The novel HLA-associated mutations identified in this study encourage us to acquire more extensive information about the viral dynamics of HLA-associated amino acid polymorphisms in a given population as effective CTL vaccine targets.

## Introduction

Accumulating evidence indicates that cytotoxic T lymphocytes (CTLs) play a central role in controlling human immunodeficiency virus (HIV) replication *in vivo*, and a number of CTL-inducing vaccines have been developed [1], [2]. All trials of CTL-inducing vaccines against HIV have been unsatisfactory including the most recent trial conducted in Thailand [3], [4], [5]. Genetic polymorphisms in the human leukocyte antigens (HLAs) are key factors contributing to the complexity of developing CTL-inducing vaccines [6], [7]. HLA class I molecules play a critical role in defining the epitopes of CTLs, which probably influence their antiviral efficacy. The extraordinary capacity of this virus to generate genetic diversity is another important factor contributing to this complexity. To date, 13 prototype HIV clades and 43 circulating recombinant forms have been described worldwide and HIV diversity appears to be increasing as the infection spreads [8].

Once the virus infects a host, it rapidly evolves and evades the host cellular immune response. Viral adaptation to the HLA-restricted immune response and the selection of viral mutations associated with the loss of the antiviral immune response have been described in both acute and chronic HIV-1 infections at the individual level [9], [10]. Recently, viral adaptations to HLA have also been reported at the population level [11], [12]. Therefore, there is a growing concern that HIV may evolve to reduce the availability of key CTL epitopes that are associated with the control of HIV infection at the population level. This in turn would greatly affect the clinical outcomes of HIV/AIDS. Therefore, these associations are becoming increasingly important for effective CTL-based vaccine strategies.

Several studies have attempted to define HLA-associated mutations in a given population using a large number of HIV genome sequences and to determine their influence on clinical outcomes [13], [14]. These studies have identified HLA polymorphisms in the HIV-1 Gag protein and this association continues to be reinforced [15]. However, most information has been derived from studies of subtype-B-HIV-infected Caucasian and subtype-C-HIV-infected African populations, and very little information is available on the CRF01_AE virus, the predominant clade circulating in southeast Asia [16], [17].

Therefore, in this study, we investigated the amino acid variations in the HIV-1 CRF01_AE Gag protein among HIV-1-infected people with known HLA alleles in Thailand, with the primary objective of identifying the amino acid mutations associated with the host HLA class I types and their influence on clinical outcomes. Moreover, because our cohort included dozens of discordant couples (viral transmission pairs), we took advantage of this point and further analyzed the fate of these HLA-selected mutations after transmission to HLA-unmatched recipients.

## Methods

### Ethical statement

This study was approved by the Ethics Committee of the Thai Ministry of Public Health and was conducted in accordance with the set guidelines for research. All patients provided their written informed consent for the collection of the samples and their subsequent analysis.

### Population and samples

We recruited 116 chronically HIV-1-infected Thai couples (219 patients in total) at a government referral hospital in northern Thailand between 6 July 2000 and 15 October 2002. The cohort has been described in detail elsewhere [18]. We obtained two sequential blood samples from each patient, with an interval of 6–27 months (mean interval 19.75 months, mode 24 months) between the two collections. The majority of patients were naïve to antiretroviral therapy, except for 27 individuals who were receiving treatment with single or dual nucleoside reverse transcriptase inhibitors. However, no patient was receiving highly active antiretroviral therapy. The median (interquartile range, IQR) CD4^+^ cell count in our study population was 163 (23, 370) cells/µL, and the median (IQR) plasma viral load was 5.20 (4.54, 5.63) log_10_ RNA copies/mL. Peripheral blood mononuclear cells (PBMCs) were separated with a commercially available cell-separation tube (CPT® Cell Preparation Tube with Sodium Citrate, BD, Franklin Lakes, NJ, USA) and used in this study.

### HLA class I typing

Genomic DNA was extracted from patient PBMCs with the QIAamp DNA Mini Kit (Qiagen, Hilden, Germany), according to the manufacturer's instructions. HLA class I typing for the A and B loci was performed using a PCR microtiter plate hybridization method (*WAKFlow*® HLA typing kit) (Wakunaga Co. Ltd., Hiroshima, Japan), according to the manufacturer's instructions. For statistical analysis, each HLA allele of each individual was assigned a two-digit designation.

### PCR amplification of HIV *gag* and sequencing

Genomic DNA was extracted from patient PBMCs as described above, and nested PCR was performed. First, the 9.1-kb nearly full-length HIV genome was amplified using Takara *LA Taq* DNA polymerase (Takara, Shiga, Japan) and the following primers, which bind to both the long terminal repeat (LTR) regions of the HIV genome: sense outer primer, MSF12b 5′-AAATCTCTAGCAGTGGCGCCCGAACAG-3′, and antisense outer primer, OFMR1 5′-TGAGGGATCTCTAGTTACCAGAGTC-3′. The PCR conditions were as follows: melting at 95°C for 5 min; 30 cycles each of 95°C for 10 s, 65°C for 30 s, and 68°C for 8 min; and a final extension at 68°C for 7 min. The 1.7-kb fragment containing the entire *gag* gene was amplified from the first round PCR product using Qiagen *Taq* DNA polymerase (Qiagen): sense inner primer, Gag-F1 5′-TCTCGACGCAGGACTCGGCTTGCT-3′, and antisense inner primer, Gag-R2 5′-CCTCCAATTCCCCCTATCATTTTTGG-3′. The thermocycling conditions for the second round of PCR were as follows: melting at 95°C for 2 min; 30 cycles each of 95°C for 30 s, 60°C for 30 s, and 68°C for 90 s; and a final extension at 68°C for 7 min. The PCR product was analyzed by gel electrophoresis. The appropriate PCR products were directly sequenced by Macrogen Inc., Korea.

### Sequence analysis

The HIV nucleic acid sequences were analyzed to identify their subtypes, using the RIP 2.0 software (http://www.hiv.lanl.gov/content/sequence/RIP/RIP.html). All CRF01_AE sequences were submitted to GenBank (accession number GU458430–GU458799). Only the sequences that included the complete *gag* open reading frame were selected for sequence analysis. The sequences were aligned and translated using the MEGA 3.1 software [19]. A consensus sequence was created from the most abundant amino acid at each position in the cohort. HIV transmission between spouses was confirmed by constructing a neighbor-joining phylogenetic tree using the entire *gag* nucleotide sequences derived from the whole sample. If the viruses derived from a husband and wife clustered on the same branch, the couple's viruses were regarded as having a common ancestor, implying that the virus was transmitted between them. On this basis, we identified 68 such couples. Each member of the remaining couples was considered to be infected with virus distinct from that infecting his/her spouse. The direction of transmission was determined by in-depth interviews with field workers. The associations between the sequence polymorphisms and the HLA types were analyzed with Fisher's exact test with a 95% confidence interval (CI), using only the patients who were source of virus in the couples (index cases), and was limited to the HLA alleles shared by at least five subjects to ensure sufficient statistical power. Amino acids that were identical to the consensus sequence were considered to be “dominant” amino acids, and any difference from the consensus sequence was classified as “non-dominant”. An amino acid position was declared an “HLA-associated variable site” if a significant HLA association was identified in the sequence at both times of sample collection. The HLA-associated variable site was mapped in relation to the best-defined CTL epitopes published in the Los Alamos HIV Databases [http://www.hiv.lanl.gov/content/sequence/HIV/mainpage.html, accessed Dec. 2009].

For detecting adaptive evolution in protein-coding sequences under natural selection in the population, the branch lengths and nucleotide substitution rate parameter was estimated to approximate the analogous parameters of the codon model. The MG94 codon model, which estimates synonymous and non-synonymous rate independently for every amino-acid, was performed. The estimating site-by-site variation rate was evaluated by single likelihood ancestor counting (SLAC) and fixed-effected likelihood (FEL) methods. The adaptive evolution study was done by HyPhy 2.0 software [20], [21].

### Statistical analysis

All statistical analyses were performed with Excel 2007. Fisher's exact test with a 95% CI was used to detect HLA-associated dominant or non-dominant sites, and Spearman's correlation test was used to determine the number of HLA-associated non-dominant sites and for the viral load correlation analysis. We also used one-way ANOVA to test the differences in viral load among the T242X mutations with or without compensatory mutations in the HLA_B*57/*58-positive or -negative groups.

## Results

### Sequencing results, study population, and HLA allele frequencies

**Figure 1:**
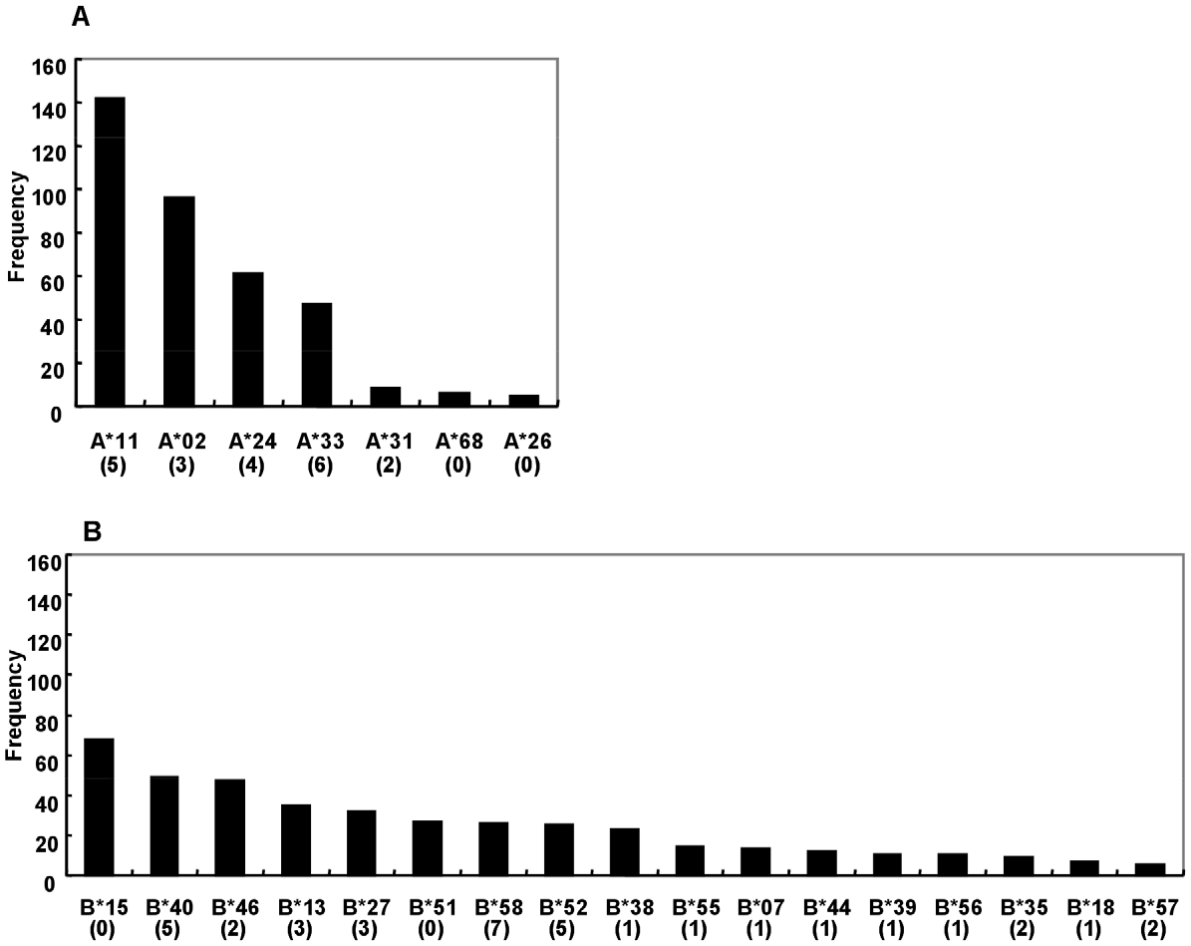
HLA_A and HLA_B allele distributions among patients. HLA allele distributions: the frequencies of HLA_A and _B alleles occurring in at least five or more individuals are shown in (A) and (B), respectively. The number of HLA-associated amino acid variations is indicated in brackets under each HLA allele.

By subtype analysis, nine individuals were found to be infected with subtype B or a CRF01_AE/subtype B recombinant form of virus, so they were excluded from further analysis. Then, 370 CRF01_AE Gag sequences were determined in the 209 and 161 samples at the first and second time points, respectively, obtained from 219 individuals (116 couples). The numbers of CRF01_AE-infected individuals carrying specific HLA class I alleles are shown in Figure 1. The most frequent HLA_A allele was A*11, followed by A*02, A*24, and A*33. The allele B*15 was the most frequent HLA_B allele, followed by B*40, B*46, and B*13. Clearly, the HLA distribution in Thailand differs from those in North American and African countries. We also analyzed the linkage disequilibrium. Strong linkage was found between A*33 and B*58 (p = 1.39×10^−12^), as previously reported elsewhere [22], [23].

### HLA-associated amino acid variations

To identify HLA-associated amino acid variations, we analyzed the Gag amino acid sequences in relation to the HLA types. Phylogenetic analysis identified 68 couples in which the CRF01_AE virus transmission between the spouses was confirmed (Figure S1). In the remaining couples, the spouses were considered to be infected with distinct viruses. To minimize the lineage effect that might result from sampling viruses from concordant couples, we included only one spouse from each couple in the analysis. After removing the contact cases from these 68 concordant couples, 144 first samples and 122 second samples were used for further analysis. We found 44 amino acid site variations (among the known 498 amino acid positions) in the Gag region. All these variations showed statistically significant associations with some of the HLA types (*p*<0.05) and these are described below.

In total, 56 associations between the HLA types and amino acid variations were identified: 20 associations with five HLA_A alleles (A*02, *11, *24, *31, and *33) and 36 associations with 15 HLA_B alleles (all except B*15 and *51). Seventeen positions (23 associations) were in p17, 16 positions (21 associations) in p24, and the remaining 11 positions (12 associations) were in p2/7/1/6 (Table 1). The associations in p17 were restricted more by HLA_A alleles (13/23 associations), whereas the associations in p24 were restricted more by HLA_B alleles (16/21 associations).

**Table 1:** HLA-associated amino acid variations.

Part	Position	Selection*	Restricted HLA	HLA	non-dominant	dominant	Odds rario	95% CI range	p value(Fisher)	Reported CTL epitope
p17	V7X	Pos	B*44	+	5	6	5.69	1.56	0.0132	
				−	17	116		20.69		
	S9X	n.s	B*13	+	12	7	22.10	6.98	8.98E-08	
				−	9	116		69.98		
	K18X	n.s	A*33	+	1	36	0.10	0.01	0.00488	
				−	24	83		0.74		
	R30X	Pos	A*11	+	52	36	2.60	1.30	0.010045622	**(Flanking) KIRLRPGGK**
				−	20	36		5.20		
			A*24	+	10	35	0.17	0.08	1.10356E-05	A*2402: KYKLKHIVW
				−	62	37		0.38		
	V35X	n.s	B*52	+	2	13	19.69	1.67	0.0282	
				−	1	128		232.20		
	T53X	Pos	A*02	+	16	51	4.52	1.56	0.004090448	A2: GLLE**S**SEGC
				−	5	72		13.12		
	A54X	Pos	A*02	+	12	55	8.18	1.76	0.00332653	A2: GLLES**S**EGC
				−	2	75		38.05		
	S66X	Pos	B*18	+	4	1	28.71	3.03	0.001572746	
				−	17	122		272.17		
	L75X	Pos	A*33	+	7	30	3.33	1.08	0.04825	
				−	7	100		10.26		
			B*57	+	2	1	21.50	1.81	0.02502	(Flanking) B57: RSLYNTVATLY
				−	12	129		254.77		
			B*58	+	7	13	9.00	2.73	0.000653882	(Flanking) B58: RSLYNTVATLY
				−	7	117		29.71		
	K76X	Pos	A*02	+	7	60	0.33	0.13	0.01934	(Flanking) A*02: SLYNTVATL
				−	20	57		0.85		
	F79X	Pos	A*24	+	6	39	0.25	0.10	0.003083254	(Flanking) A24: LYNTVATL
				−	38	61		0.64		
	T81X	Pos	B*58	+	8	12	4.84	1.70	0.004583808	B58: RSLYN**T**VATLY
				−	15	109		13.77		
	V82X	Pos	A*24	+	24	21	4.51	2.10	0.000153966	(Flanking) A24: LYNTVATL
				−	20	79		9.69		
			B*58	+	11	9	3.37	1.28	0.0171	B58: RSLYNT**V**ATLY
				−	33	91		8.86		
	V83X	Pos	A*11	+	22	66	0.09	0.04	2.48E-10	**IATLWCVHQR**
				−	44	12		0.20		
			A*24	+	30	15	3.50	1.67	0.001063416	A24: LYNTV**A**TL
				−	36	63		7.36		
	E93X	n.s	B*40	+	21	14	13.36	5.33	8.96E-09	B*4001: I**E**IKDTKEAL
				−	11	98		33.52		
	I104X	Pos	A*11	+	64	24	4.44	2.17	5.18849E-05	A11: K**I**EEEQNKSK
				−	21	35		9.10		
			B*27	+	4	9	0.27	0.08	0.0392	
				−	81	50		0.94		
	S125X	n.s	A*11	+	15	73	3.63	1.00	0.0419	
				−	3	53		13.18		
p24	M186X	Purify	B*35	+	1	4			0.0347	
				−	0	139				
	A196X	n.s	B*38	+	4	13	38.77	4.03	0.000641253	B38: GHQ**A**AMQML
				−	1	126		373.21		
	E203X	n.s	B*52	+	4	11	6.34	1.60	0.01658	(Flanking) B52: HQAAMQMLK
				−	7	122		25.06		
	I223X	n.s	B*13	+	4	15	6.40	1.55	0.0181	(Flanking) B13: GQMREPRGSDI
				−	5	120		26.48		
	M228X	n.s	B*13	+	9	10	5.35	1.91	0.002052971	B13: GQ**M**REPRGSDI
				−	18	107		14.98		
	T242X	Pos	A*33	+	14	23	12.42	4.06	2.88923E-06	
				−	5	102		37.94		
			B*46	+	0	34	0.00		0.007061705	
				−	19	91				
			B*57	+	3	0			0.001988329	B*5701, 5703: TS**T**LQEQIGW
				−	16	125				
			B*58	+	15	5	90.00	21.75	6.06E-13	B*5801: TS**T**LQEQIGW
				−	4	120		372.40		
	G248X	n.s	A*33	+	8	29	3.94	1.32	0.0238	
				−	7	100		11.78		
			B*58	+	8	12	11.14	21.75	0.000120432	B*5801: TSTLQEQI**G**W
				−	7	117		372.40		
	P255X	Pos	A*11	+	20	68	7.94	1.78	0.001603328	
				−	2	54		35.48		
	V280X	Pos	B*46	+	12	22	14.45	4.26	4.9055E-06	
				−	4	106		49.02		
	S281X	n.s	B*52	+	10	5	21.45	6.22	9.32E-07	B*5201: RMYSPT**S**I
				−	11	118		74.04		
	R286X	n.s	B*52	+	7	8	13.23	3.82	0.000122838	(Flanking)B*5201: RMYSPTSI
				−	8	121		45.79		
	D295X	Purify	B*39	+	2	7	38.29	3.09	0.010144785	
				−	1	134		474.84		
	T310X	n.s	A*33	+	13	24	3.92	1.61	0.005101645	A*33 motif: VDRFYKLTRAEQA**S**
				−	13	94		9.54		
			B*58	+	8	12	3.93	1.41	0.011165919	B*5801: QA**T**QDVKNW
				−	18	106		10.94		
	N315X	Purify	A*33	+	8	29	5.63	1.71	0.004581687	(Flanking)A*33: VDRFYKLTRAEQAS
				−	5	102		18.52		
	T348X	n.s	B*35	+	1	4			0.0347	
				−	0	139				
	S357X	Purify	B*07	+	2	8	10.92	1.59	0.039	B*0702: GP**S**HKARVL
				−	3	131		74.94		
p2	R387X	Pos	A*31	+	4	2	8.22	1.43	0.0197	
				−	27	111		47.26		
			B*27	+	6	7	3.63	1.12	0.0345	
				−	25	106		11.76		
p7	HXB2 R403X	Pos	A*31	+	2	4	16.75	2.34	0.020250589	A*3101: LARNCRAPRK
	(R401X)			−	4	134		119.78		
p6	HXB2 P453X	Pos	B*55	+	6	3	18.77	4.19	0.000158687	
	(P451X)			−	13	122		84.06		
	HXB2 T456X	Pos	B*56	+	5	3	5.89	1.33	0.02086	
	(T454X)			−	30	106		26.07		
	M463X	n.s	B*27	+	6	7	5.38	1.62	0.008955598	
				−	18	113		17.84		
	HXB2 Q476X	n.s	B*52	+	4	11	15.27	3.03	0.002287463	
	(Q473X)			−	3	126		77.09		
	HXB2 E480X	Purify	B*40	+	11	24	7.87	2.65	0.000183624	B*4001: K**E**LYPLTSL
	(E479X)			−	6	103		23.39		
	HXB2 L483X	n.s	B*40	+	22	13	4.90	2.18	0.000166254	B*4001: KE**L**YPLTSL
	(H480X)			−	28	81		11.00		
	HXB2 485X	n.s	B*40	+	7	28	3.64	1.18	0.0424	B*4001: KELY**P**LTSL
	(P482X)			−	7	102		11.25		
	HXB2 485X	Purify	B*40	+	7	28	3.64	1.18	0.008631568	B*4001: KELY**P**LTSL
	(P483X)			−	7	102		11.25		
	HXB2 T487X	n.s	B*58	+	7	13	6.88	2.20	0.0018202	B58: L**A**SLRSLF
	(V485X)			−	9	115		21.56		

Foot note for Table 1: *Pos. positive selection, n.s. not significant; P483X was an insertion mutation.

The number of HLA-associated amino acid variations did not necessarily correlate with the frequency of the allele. More than five amino acid variations were associated with B*58 and B*52, despite the relative infrequency of these alleles, whereas no variation was significantly associated with one of the most frequent alleles, B*15 (Figure 1B). Among the 56 HLA-associated amino acid variations, 49 (87.5%) were selected by non-dominant amino acids in the presence of the specific HLA type. The remaining seven (12.5%) variations were selected by dominant amino acids in the presence of a specific HLA type. Six amino acid variations caused by negative selection were located in p17, whereas only one was located in p24. Dominant amino acid selection was always associated with frequent HLA alleles: five variations were associated with A alleles (A*11, A*02, A*24, or A*33) and two were associated with B alleles (B*46 or B*27).

We also found that 35 (62.5%) HLA-associated amino acid variations were located within or adjacent to the best-defined HIV-specific CTL epitopes, restricted by the relevant HLA allele [24] (Table 1). Some HLA-associated amino acid variations were located at anchor positions of binding peptides: A*24-associated F79X, B*40-associated E93X, and B*58-associated V485X (Table 1). Odds ratios were widely variable, ranging from 2.60 to 90.0, with a median (IQR) of 7.87 (4.48, 13.3). The odds ratio was highest by far at B*58-associated T242.

The codon-based analysis revealed a large number of significant selection sties in the Gag protein, mostly purifying selection; among the 498 Gag amino acid positions, 270 (54.2%) sites and 52 (10.4%) sites were identified by either SLAC or FEL method as purifying selection and positive selection sites, respectively (Table S1). Interestingly 19 (36.5%) out of the 52 positive selection sites located at the sites of HLA-associated amino acid variations, whereas only 6 (2.2%) out of 270 purifying selection sites located at the sites of HLA-associated amino acid variations (Table 1). This implies that HLA-pressure is one of major factors driving the positive amino acid selection among Gag protein.

### Associations between numbers of HLA-associated amino acid variants and clinical outcomes

**Figure 2:**
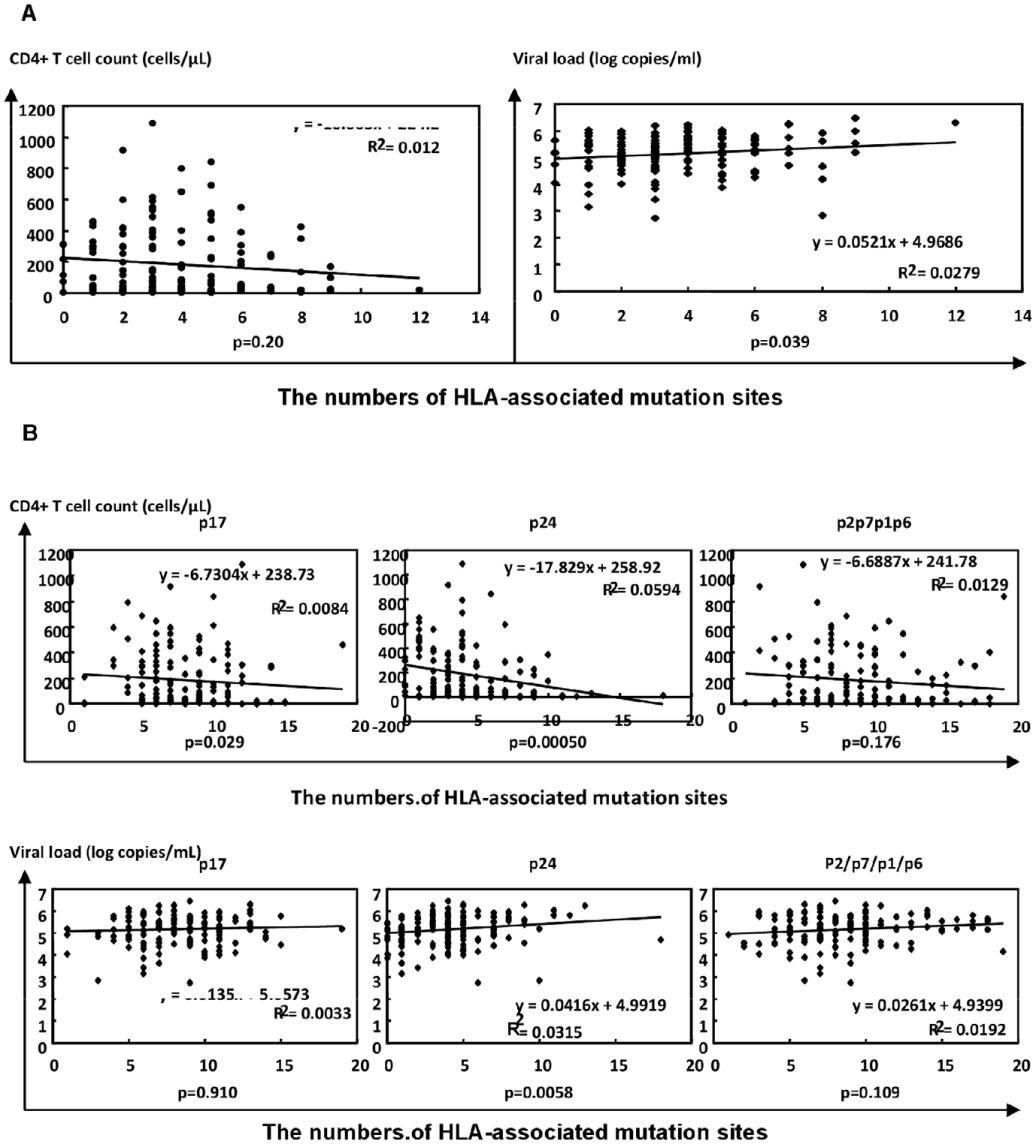
Associations between numbers of HLA-associated mutation sites and CD4 cell count or viral load. A regression line is drawn in each graph: (A) shows the relationships with HLA-associated amino acid variations in the whole Gag sequence, and (B) shows the relationships with amino acid variations in p17, p24, and p2/p7/p1/p6.

After defining the HLA-associated amino acid variation sites in CRF01_AE in the analysis described above, we counted the numbers of HLA-associated variations in autologous viral sequences for each patient, and plotted them on the X axis, and plotted the plasma viral loads and CD4^+^ cell counts on the Y axis. We found significant associations between the numbers of HLA-associated amino acid variations and the CD4^+^ cell counts or viral loads. Patients with a higher number of HLA-associated amino acid variants tended to have a higher plasma viral load and lower CD4^+^ cell counts (Figure 2A). We further analyzed these associations according to the subregions of Gag in which the variations occurred. Intriguingly, these correlations were mainly driven by the associations with variations in the p24 region (Figure 2B).

### Amino acid variations in a recipient host with different HLA alleles

**Figure 3:**
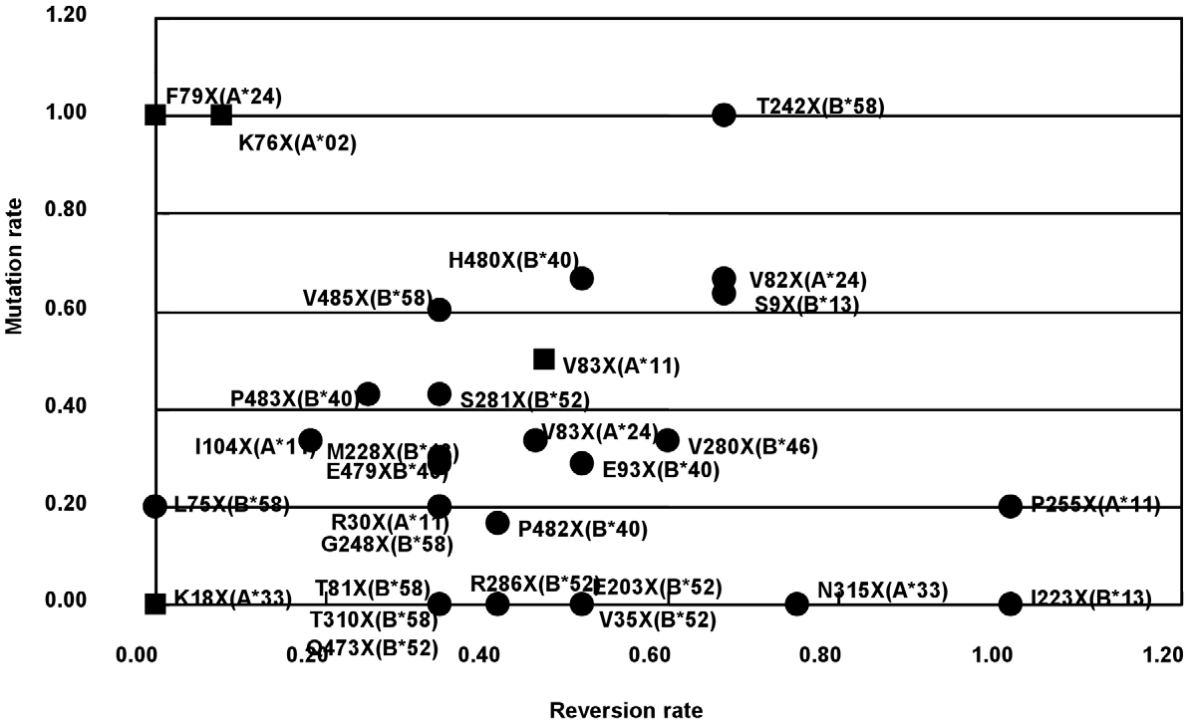
Rates of reversion and mutation for each HLA-associated amino acid variant. HLA-associated mutation and reversion rates after viral transmission to contact spouses with distinct HLA profiles were calculated and plotted. Amino acid variations selected by non-dominant amino acid (•) and dominant amino acid (▪) are shown.

With in-depth interviews conducted by designated field workers, the index and contact cases were determined among the 65 concordant couples. Looking at the viral sequences in a pairwise manner, we noted that the frequencies of *de novo* HLA-associated mutations and reversions after viral transmission to contact cases with distinct HLA profile varied considerably, depending on the amino acid positions involved. Mutations and reversions of each HLA-associated amino acid variant were studied whenever data for at least five couples were available. When the virus was transmitted to a contact case with a different HLA environment, as confirmed by sequencing, the rate of reversion or mutation for each HLA-associated amino acid variant was calculated and was plotted on a scatter graph (Figure 3). To avoid overestimation of the mutation or reversion rate, we counted only HLA-associated sites with *p* values of <0.01 with a 99% CI and with a denominator of more than one when we calculated their rates. In total, 30 HLA-associated amino acid variation sites were listed. For instance, at the S9X site restricted by B*13, which was selected by non-dominant amino acid, the mutation rate was calculated as 5/11 ( = 0.45), five S9T–B*13-positive contact cases divided by 11 S9S–B*13-negative index cases. Its reversion rate was calculated as 4/6 ( = 0.67), four S9S–B*13-negative contact cases divided by six S9T–B*13-positive index cases (see supplementary data for details of the mutation and reversion sequence variations, Table S2). For dominant amino acid selection sites, the mutation rate and reversion rate were calculated in the opposite way. At the K76X site restricted by A*02, the mutation rate was calculated as 3/3 ( = 1.0), three K76K–A*02-positive contact cases divided by three K76R–A*02-negative index cases. The reversion rate was calculated as 1/13 ( = 0.077), one K76R–A*02-negative contact case divided by 13 K76K–A*02-positive index cases. The average reversion rate was 0.42 and the average mutation rate was 0.33. There was a rough inverse relationship between the reversion and mutation rates. P255X (A*11) and I223X (B*13) scored reversion rates of 1.00 and both had low mutation rates. Conversely, F79X (A*24), K76X (A*02), and T242X (B*58) had mutation rates of 1.00 and the former two had low reversion rates. Interestingly, T242X (B*58) was outstanding in that both its mutation rate and reversion rate were very high. This indicates that the rate of accumulation of CTL escape mutations in a given population varies considerably among mutations and restricting HLA types.

### T242N mutations

As described above, T242X had a high reversion rate. The vast majority of T242X mutations were T242N, known as an escape mutation from CTL (TSTLQEQIGW: TW10), restricted by the protective HLA alleles B*57 and B*5801 in the setting of clade B and C infections. This mutation emerges almost universally in B*57/*5801-positive subjects. Several studies have demonstrated that the T242N substitution affects viral replicative fitness *in vitro* and it is believed to contribute to the protective effect of these alleles against the progression of HIV disease [25], [26]. Moreover, several mutations within the cyclophilin A binding loop, such as H219 and M228, have been shown to compensate to some extent for the reduced viral replicative capacity caused by T242N [27], [28]. However, the roles of T242N and the compensatory mutations in CRF01_AE infections are unknown. HLA_B*5801 is known to present the same epitopes as B*57 [29], and our unpublished data indicate that the vast majority of B*58 alleles in Thailand are B*5801. There were no statistically significant differences in the plasma virus loads of the B*57/*58-positive and -negative populations in our cohort (data not shown). Five of the 23 B*57/*58-positive subjects did not carry T242X, and there was no statistically significant difference in their plasma viral loads or CD4 cell counts, i.e., in terms of the presence or absence of T242X in these patients (data not shown). We then stratified the B57*/*58-positive patients with T242X according to the presence/absence of the described compensatory mutations. Interestingly, we found that B*57/*58-positive patients with the compensatory mutations had significantly higher viral loads and lower CD4 cell counts than those without the compensatory mutations (Figure 3), indicating that the proposed mechanism of virus attenuation by the escape mutation and its restoration by the compensatory mutations at the B*57/*5801 TW10 epitope is applicable in the context of CRF01_AE infections.

### T242N mutations and transmission

It was recently reported that the transmission of viruses with attenuating CTL escape mutations, particularly T242N from B*57-restricted CTL, is associated with better early clinical outcomes in HLA-unmatched recipients [30], [31]. However, the long-term effects of the transmission of these viruses to HLA-unmatched recipients remain unknown. We summarized the amino acid variations around the TW10 epitope in B*57/*58-negative contact cases who had contracted the virus from B*57/*58-positive spouses and their clinical features (Table 2). Only two B*57/*58-negative spouses carried the T242N mutation at the time of sampling. Both had very high CD4 cell counts of >500 cell/µL and very low viral loads of less than 10^4^ copies/mL, which is in distinct contrast to the remaining six B*57/*58-negative spouses who lacked T242N (median plasma viral load, 5.39 log copies/mL), and supports the results of the recent study by Chopera et al. [28]. However, because the T242N escape mutation is known to emerge within the first three months of infection in B*57-positive subjects [32], it is unlikely that these six contact cases had acquired the wild-type T242 virus, but instead, the transmitted T242N probably reverted after its transmission to these recipients. These data suggest that the majority of the recipients from B*57/*58-positive donors do not receive the benefit conferred by the transmission of the attenuated virus after many years of infection, although we did not know the duration of the infection in each patient in the present study. We identified three other patients without the B*57/B*58 alleles who carried viruses with T242N, and they all had very low viral loads of less than 10^4^ copies/mL (data not shown). We presume that they contracted the virus from B*57/B*58-positive patients, although we could not identify their index cases in our study population. Taken together, these results imply that the transmission of CTL-selected attenuated viruses might confer a survival advantage on HLA-unmatched recipients, at least during the early stage of infection, and that this advantage is not limited to infection with a particular clade of virus. However, this effect may not be retained for an extended period of time.

**Table 2:** Clinical status and amino acid variations around TW10 and compensatory mutations (H219, I23, and M228) for T242N in eight couples with B*57/B*58 index cases.

Sex	AIDS	CD4 (/µl)	VL (log copies/mL)	HLA_A1	HLA_A2	HLA_B1	HLA_B2	H219	I223	M228	D235	I236	A237	G238	T239	T240	S241	T242	L243	Q244	E245	Q246	I247	G248	W249	M250	T251	N252	N253	P254
M	No	11	5.35	A*11	A*33	B*40	B*57	-	-	-	-	-	-	-	-	-	-	N	-	-	-	-	-	-	-	-	-	G	-	-
F	No	311	5.37	A*02	A*11	B*15	B*15	-	-	-	-	-	-	-	-	-	-	-	-	-	-	-	-	-	-	-	-	G	-	-
M	No	13	5.21	A*24	A*33	B*52	B*58	-	-	-	-	-	-	-	S	-	-	N	-	-	-	-	-	-	-	-	-	S	-	-
F	No	196	5.41	A*11	A*34	B*15	B*56	Q	-	-	-	-	-	-	-	-	-	-	-	-	-	-	V	-	-	-	-	S	-	-
M	Yes	3	5.92	A*02	A*33	B*40	B*58	Q	-	I	-	-	-	-	-	-	-	N	-	-	-	-	-	T	-	-	-	-	-	-
F	No	627	3.71	A*02	A*11	B*13	B*38	-	-	I	-	-	-	-	-	-	-	N	-	-	-	-	-	A	-	-	-	-	-	-
M	Yes	230	6.28	A*11	A*11	B*58	B*58	Q	-	I	-	-	-	-	-	-	-	N	-	-	-	-	-	A	-	-	-	-	-	-
F	No	284	5.63	A*11	A*11	B*40	B*51	-	-	-	-	-	-	-	-	-	-	-	-	-	-	-	-	-	-	-	-	S	-	-
M	Yes	9	5.72	A*11	A*33	B*15	B*58	Q	-	I	-	-	-	-	-	-	-	N	-	-	-	-	-	A	-	-	-	-	-	-
F	No	195	5.76	A*11	A*24	B*40	B*51	Q	-	-	-	-	-	-	-	S	-	-	-	-	-	-	-	-	-	-	-	G	-	-
M	No	204	5.65	A*11	A*33	B*38	B*58	-	-	I	-	-	-	-	-	-	-	-	-	P	-	-	-	A	-	I	-	-	-	-
F	No	11	5.31	A*02	A*11	B*35	B*40	-	-	-	-	-	-	-	-	-	-	-	-	-	-	-	-	-	-	-	-	S	-	-
M	No	688	4.35	A*24	A*33	B*15	B*58	-	-	-	-	-	-	-	-	-	-	N	-	-	-	-	-	-	-	-	-	-	-	-
F	No	576	3.91	A*11	A*24	B*15	B*52	-	-	-	-	-	-	-	-	-	-	N	-	-	-	-	-	-	-	-	-	-	-	-
M	Yes	19	6.32	A*11	A*33	B*56	B*58	Q	-	-	-	-	-	-	-	-	-	N	-	-	-	-	-	-	-	-	-	-	-	-
F	No	441	4.98	A*24	A*24	B*18	B*27	-	-	-	-	-	-	-	-	-	-	-	-	-	-	-	-	-	-	-	-	-	-	-

## Discussion

This is the first published study that systematically analyzes variations in the Gag sequence and their associations with HLA in HIV-1 CRF01_AE infections. We identified 56 amino acid variations at 44 amino acid positions, which were significantly associated with a particular HLA class I type. We found that a substantial number of HLA-associated amino acid variations appeared to be unique to this CRF01_AE-infected Thai population. However, despite these distinct variants, we confirmed that the capsid protein (p24) is probably the preferred target of CTLs in CRF01_AE infections. We also found that the reversion rate of these putative CTL escape mutations upon transmission to HLA-unmatched recipients varies considerably, suggesting that the rate of accumulation of CTL escape mutations in a given population differs substantially between mutations. Our data also suggest that the transmission of CTL-selected attenuated viruses is likely to confer a survival benefit on HLA-unmatched recipients, at least during the early days of the infection.

These associations between HLA and viral sequences can be explained in several ways. The majority of associations are probably attributable to specific mechanisms by which the virus escapes from HLA-restricted, HIV-specific CTLs, such as loss of peptide binding, loss of T-cell receptor recognition, and/or changes in peptide processing and presentation [1]. In fact, we found that two thirds of the HLA-associated variations were located within known CTL epitopes or their flanking regions. However, most previously reported CTL epitopes were identified in studies of other clades, indicating that these CTLs are probably cross-clade CTLs. Because studies of CTL epitopes in CRF01_AE infections are limited, we believe that several other mutations may occur in clade-specific CTL epitopes that have not yet been reported. Our current work on CTL epitope mapping using overlapping CRF01_AE Gag peptides has identified several new CTL epitopes, and at least two amino acid mutations have been found within the newly identified CRF01_AE Gag CTL epitopes (data are in preparation). Linkage disequilibrium can also explain the associations between HLA and viral sequences. There is a strong association between HLA_A*33 and _B*58 [22], [23]. The A*33-associated T242X and G248X mutations are widely known to be selected by HLA_B*57/*5801 [26], so they are likely to be reflected in the linkage disequilibrium with B*58. Some HLA-associated mutations may also be part of the structural and functional compensatory mechanism underlying the development of primary CTL escape mutations, which can arise at sites considerably remote from the relevant epitopes [30].

This study has relatively low statistical power compared with previous studies. However, one of its strong aspects is that we identified a substantial number of HLA-associated amino acid variations that appear to be unique to a CRF01_AE-infected Thai population. Many of these variations do not appear in the list of HLA-associated mutations identified in subtype-B infections in over 1,500 subjects [15], and this cannot be explained by the relatively low statistical power of the present study. For instance, mutations such as S9X, V280X, and P453X had convincingly strong associations with HLA_B*13, _B*46, and _B*55, respectively (*p*<0.001; Table 1). However, none of these associations have been noted in subtype B, suggesting that there are a number of unidentified CTL pressures on Gag in the context of CRF01_AE infections in the Asian population. This indicates that an extensive search for CTL epitopes in various clades is warranted to facilitate the development of globally effective CTL vaccines.

Several publications have suggested that the Gag CTL response, as measured by interferon γ ELISPOT, has the most profound effect on the clinical outcome in subtype B and subtype C infections. Responses to the capsid protein are also likely to be most crucial in the containment of viral replication *in vivo* [31]. In this study, we have shown that variations in the capsid protein (p24) had the strongest association with viral load and CD4 cell count, indicating that regardless of the HIV clade, the p24 capsid is the most preferred target of HIV-1-specific CTLs. Therefore, p24 may be one of the important targets for effective CTL-based vaccines.

### Reversion rates and mutation rates

When the virus is transmitted into a new host with a different HLA environment, the virus, which had already adapted to the previous host, must adapt again to the new HLA environment by reversion of the previous mutations and/or the creation of new mutations. One of the most interesting results of the present study is the variability in the reversion and mutation rates for each HLA-associated variant. We found that these rates varied considerably, depending upon the amino acid position, and that the reversion rate tended to correlate inversely with the mutation rate. It is plausible that an amino acid change with a low reversion rate tends to accumulate in the population, and that the rate of accumulation is higher if the mutation rate is higher and especially if its HLA allele is dominant. In fact, we found that all the variations selected by dominant amino acid were associated with common HLA alleles and rarely changed from the consensus sequence, even after they were transmitted to HLA-unmatched recipients. These findings suggest that the viruses circulating within the population had already adapted to the common HLA alleles, resulting in best-fit sequences that could escape from those alleles. The results of our study will increase our understanding of the influence of immune pressure on HIV and on the future direction of virus evolution. Conversely, it is also plausible that an amino acid change with a high reversion rate, presumably with a functional or structural constraint at that position, is unlikely to accumulate rapidly in the population. It will be important for future CTL vaccine development to consider whether an escape mutant will accumulate or not. Therefore, further studies of this kind are required to provide valuable insights for future vaccine design.

### T242N issues

**Figure 4:**
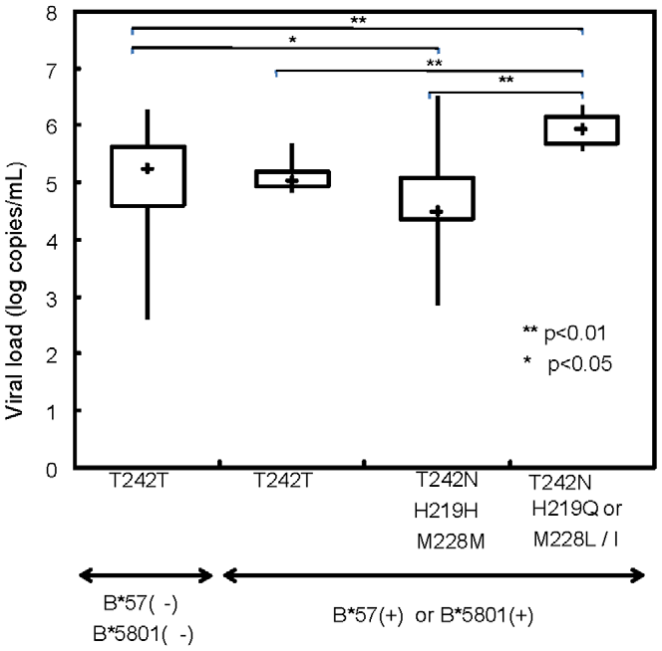
Lower plasma viral loads in B*57/*5801-positive subjects with T242N. Plasma viral loads of patients grouped by the presence or absence of B*57/*5801 with T242N with or without the compensatory H219 and M228 mutations.

This is the first report indicating that the p24 T242N escape mutation from the B*57/*58 CTL has a significant impact on the HIV-1 viral load in CRF01_AE infections and that the mutations, H219 and M228, compensate for the crippling effect of T242N. Although this is a rather predictable result because the TW10 site is conserved throughout the HIV-1 clades, it increases our insight. Two other dominant CTL epitopes within the capsid protein are restricted by B*57 (IW9: ISPRTLNAW; KF11: KAFSPEVIPMF). However, the founder virus of CRF01_AE has a well-described ‘peptide-processing mutation’ at IW9 that affects the epitope presentation on HLA class I molecules, and a well-described CTL escape mutation within KF11 [33]. In fact, 100% of the CRF01_AE sequences in our cohort carried these amino acid substitutions (data not shown; see the Methods, for the GenBank accession number), indicating that these two CTL epitopes are less likely recognized in CRF01_AE infections. Several lines of evidence indicate that TW10 is the most important target determining the viral load set point: TW10 is the earliest target during primary infection [32]; and among all the described HLA-associated mutations, T242N is the earliest escape mutation that emerges during acute infections [13]. In light of these previously reported data and the nature of the CRF01_AE sequence, it would be interesting to determine whether TW10 is sufficient to protect B*57/*5801-positive subjects from disease progression. Unfortunately, we saw no clear protective effect of B*57/*5801 in the cohort reported here. However, as shown by the low median CD4^+^ T-cell count, our cohort was substantially advanced in terms of disease progression. Therefore, the accumulated compensatory mutations might have masked the true protective effect of B*57/*5801. Supporting this explanation, we observed substantially lower plasma viral loads in the B*57/*5801-positive subjects with T242N but without the compensatory mutations (*p* = 0.012 by ANOVA; Figure 4).

We have also shown that the transmission of virus crippled by T242N might be associated with lower plasma viral loads in HLA-unmatched recipients, supporting a previous study of clade C infections [34]. However, the long-term effects of the transmission of virus attenuated by CTL escape mutations remain unknown and these contact cases should be examined longitudinally to determine whether virological escape accompanies the reversion of the T242N escape mutation.

### Limitations

One of the limitations of this study is that we had no information regarding the timing of HIV transmission within the couples, although the rate of mutation is known to depend on the time from transmission [35]. However, perhaps because the duration of marriage in our discordant couples was quite long (median of seven years), we detected negligible amino acid changes between the first and second samplings. Therefore, we believe that most of the associations between amino acid mutations and HLA alleles observed in this study occurred in chronic infections. Another limitation is that this type of analysis depends heavily on statistical power. Therefore, it is difficult to identify HLA-associated variations if the allele frequencies and rates of mutation are low. Moreover, we did not use multiple testing corrections because of the small sample size, so we must admit there would have been a substantial number of false positive results. However, the high odds ratios of some of the novel HLA-associated mutations in the context of CRF01_AE infections in this Asian population strongly encourage us to obtain more information about CTL epitopes in different geographical regions where distinct HIV clades circulating, to develop globally effective CTL-based vaccines.

Our contact cases were not incident cases. Inevitably, there is also concern that the estimated direction of viral transmission was not true. However, because the HIV epidemic in Thailand started with commercial sex workers transmitting to their male clients and then from husbands to their wives [16], and partly because our study was conducted in a hospital close to a rural community, our interviews clearly indicated which spouse displayed risk behavior for HIV infection in most couples.

In conclusion, our data suggest two points: (a) HLA-associated amino acid mutations and CTL selection pressure on the p24 antigen appear to have the most significant impact on HIV replication in this CRF01_AE infection in an Asian population; and (b) the rates of accumulation of CTL escape mutations at the population level differ substantially between escape mutations, because the reversion rates varied considerably among the HLA-associated mutations.

## Supporting Information

Figure S1 The inserted box magnifies the phylogenetic tree to show how the couples were identified.
(0.48 MB TIF)

Table S1 The codon-based analysis demonstrated selection sites in the Gag protein.
(0.28 MB XLS)

Table S2 Sequence mutation or reversion list.
(0.04 MB XLS)
