# Supplementary figures and images for: HLA-Associated Immune Pressure on Gag Protein in CRF01_AE-Infected Individuals and Its Association with Plasma Viral Load
Source: PLoS One. 2010 Jun 17;5(6):e11179. doi: 10.1371/journal.pone.0011179 (PMC2887364; doi:10.1371/journal.pone.0011179)

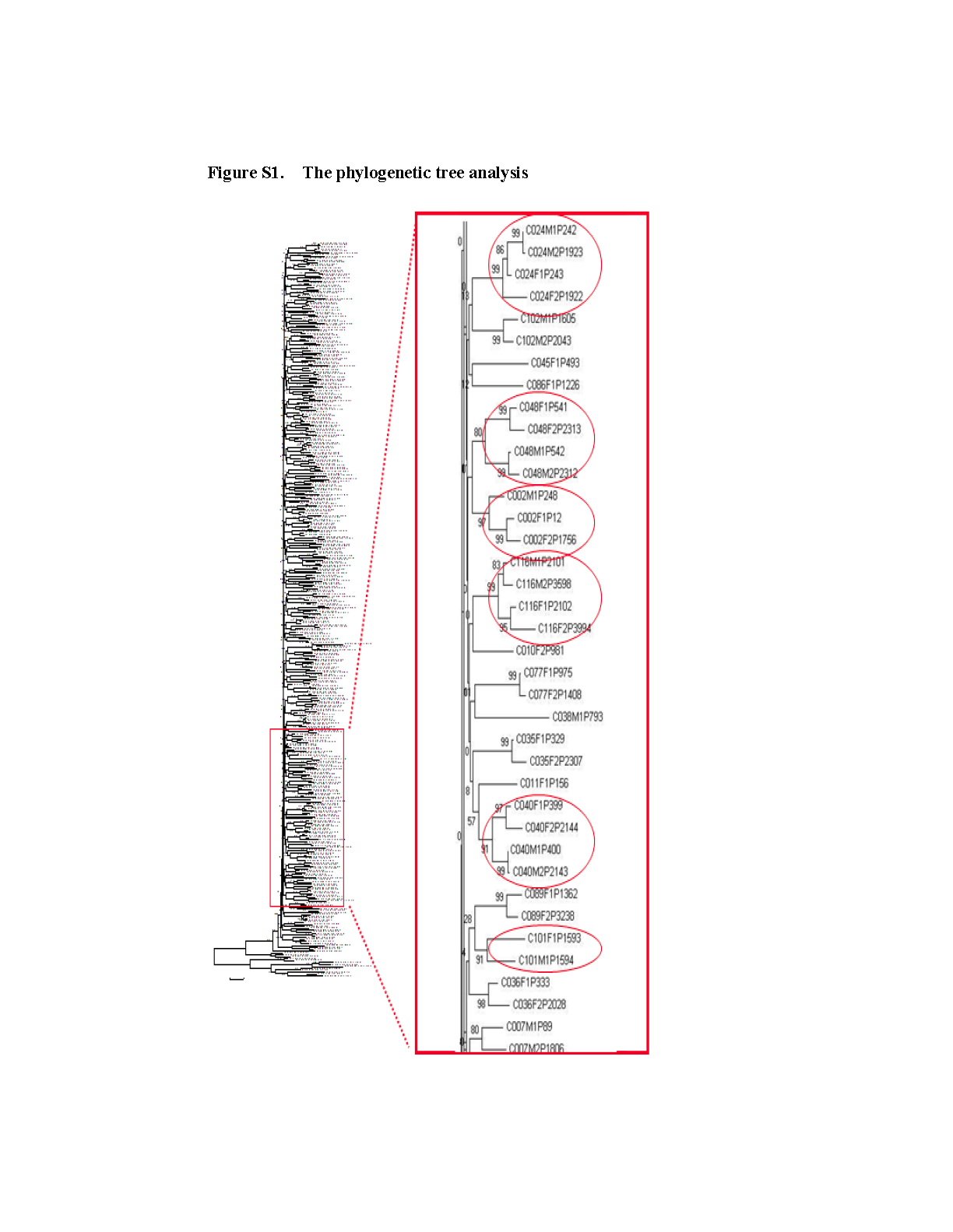

Supplement: Figure S1 — The inserted box magnifies the phylogenetic tree to show how the couples were identified. (0.48 MB TIF) [file pone.0011179.s001.tif]
